# Distribution and speciation of Cu and Zn near spring barley (*Hordeum vulgare*) roots in digested sewage sludge-amended soil

**DOI:** 10.1007/s10653-025-02482-0

**Published:** 2025-04-13

**Authors:** Jianting Feng, Ian T. Burke, Felipe E. Sepúlveda Olea, Xiaohui Chen, Douglas I. Stewart

**Affiliations:** 1 School of Civil Engineering, University of Leeds, Leeds, LS2 9JT UK; 2 Key Laboratory of Ministry of Education for Geomechanics and Embankment Engineering, Hohai University, Nanjing, 210024 Jiangsu China; 3 School of Earth and Environment, University of Leeds, Leeds, LS2 9JT UK

**Keywords:** Electron probe microanalysis, Metal speciation, Microfocus X-ray absorption near edge spectroscopy, Microfocus X-ray fluorescence, Spatial distribution

## Abstract

Risk management for agricultural use of digested sewage sludge requires better understanding of the behaviour and fate of contaminant metals in the plant root zone. A study employing rhizo-pot and plug-tray experiments was conducted to identify the zone near spring barley roots (*Hordeum vulgare*) where concentration and speciation of Cu and Zn are affected. Cu and Zn bonding environments in the root epidermis/cortex and vascular tissue were also identified. In the digested sludge-amended soil, spring barley absorbed Cu only from the immediate vicinity of the roots (<< 1 mm), but Zn was taken up from further afield (> 1 mm). In the rhizosphere Cu was predominately present as Cu(I) oxides or as Cu(II) absorbed/bonded to phosphate, whereas Zn was present as Zn(II) in inner-sphere complexes with metal oxide surfaces, as Zn(II) sulphides or Zn(II) bonded to/incorporated into carbonates. Cu taken-up by spring barley roots was largely sequestered in the root epidermis and/or cortex predominately in the coordination environments similar to those seen in the rhizosphere. Only a small proportion of the Cu was translocated into the vascular tissue (where it is in the same two bonding environments). Zn taken-up by spring barley roots was present as Zn(II) sulphides, Zn(II) absorbed to/incorporated into carbonates, or Zn(II)-organic complexes. Zn was readily translocated from roots to shoots. Better understanding of these differences in the mobility and uptake of Cu and Zn in sludge-amended agricultural soils could be used to undertake element specific risk assessments.

**Supplementary Information:**

The online version contains supplementary material available at 10.1007/s10653-025-02482-0.

## Introduction

The use of sewage sludge (a by-product of wastewater treatment) as an alternative to organic fertilisers (Asgari Lajayer et al., 2019a), for improving agricultural soil fertility, is strictly regulated in many countries due to the presence of contaminant metals (Arteaga et al., 2024; Asgari Lajayer et al., 2019b; DEFRA, 2018; EPA, 2022; Gautam et al., 2022; Hušek et al., 2023; Phung et al., 2023; Wang et al., 2020). Those regulations typically permit sludge use in agriculture only if metal concentrations in the sludge do not exceed permissible levels, and they require careful monitoring of soil metal concentrations following sludge application (Jasińska, 2018). However, these controls are being increasingly viewed as overly conservative as they do not recognise the relevance of metal speciation (which can evolve over time) on the risk assessment (Dede et al., 2023; Feng et al., 2023; Shivakumar et al., 2012; Yang et al., 2017). This may lead to an overestimation of the risk, which in turn can limit the potential of sewage sludge use in agriculture. Feng et al. (2024) therefore, investigated the two most abundant metals in sewage sludge, Zn and Cu, and determined their speciation changes in a sludge-amended soil that occurred over time and with repeated plant growth. They found that over time both Cu and Zn in the sludge-amended soil become progressively less mobile, and the transformation is slightly enhanced in the presence of plant growth.

Most previous studies of the behaviour and fate of contaminant metals in sludge-soil–plant systems have focussed on the bulk soil (at a distance from the plant root surfaces) in the plant root zone (Feng et al., 2024; Su et al., 2004; Youssef & Chino, 1988). However, plant roots can significantly influence the environment directly adjacent to them (rhizosphere) to obtain access to essential nutrients from the sludge-amended soil (Kissoon et al., 2010). As a result, environmental conditions in the rhizosphere soil differ considerably from the bulk soil (Krishnamurti et al., 1996; Pirrone et al., 2013). Thus, there is a pressing need for more research on contaminant metal behaviour in close proximity to plant roots in the sludge-amended soil, to improve the risk management when sewage sludge is used in agriculture (Lin et al., 2003).

The aim of this study is therefore to understand the changes in metal concentration and transformation of metal speciation that occur in close proximity to spring barley roots (*Hordeum vulgare*) in the digested sewage sludge-amended soil. The hypotheses are that metal concentrations and speciation will be altered in the rhizosphere due to plant root-induced chemical and microbial changes. This work primarily focuses on Cu and Zn introduced with sewage sludge applied to agricultural land as a fertiliser due to their abundance in sewage sludge, their agronomic value as essential micronutrients and their detrimental impacts at higher concentrations. The objectives are to: (1) quantify spatial variations in Cu and Zn concentrations in close proximity to plant roots; (2) characterize the transformation of Cu and Zn speciation and bioavailability in the rhizosphere due to plant growth; (3) evaluate the potential influence of digested sludge amendment on metal mobility and uptake by spring barley roots. First the zone where Cu and Zn concentrations are significantly affected by spring barley roots was investigated in a rhizo-pot system (where the plant roots and sludge-amended soil are physically separated). Then the detailed *in-situ* spatial distribution and speciation of Cu and Zn in close proximity to spring barley roots were investigated at micrometre scale in a plug-tray system using electron probe microanalysis (EPMA), microfocus X-ray fluorescence (μXRF) and microfocus X-ray absorption near edge spectroscopy (μXANES). This understanding of the behaviour and fate of contaminant metals in the plant root zone were further used to assess the risks associated with sewage sludge application in agricultural land.

## Materials and methods

### Collection and preparation of materials

#### Collection of sewage sludge and agricultural soil

Sewage sludge was collected from Esholt Wastewater Treatment Works, Leeds, UK, on 1st March 2023. It was a secondary treatment sludge that had undergone further thermal hydrolysis (165 °C at 6 bar for 30 min) prior to anaerobic digestion. The pH of this digested sludge was 7.9 and its dry solids content was 53.3 g/L. Organic matter content of this sludge was 51.1%. The digested sludge contained about 180 mg Cu and 600 mg Zn per kg dry sludge solids. The agricultural soil was collected from a working arable field at Spen Farm, Tadcaster, UK (Lat. 53.8699, Long. −1.3290) on 24th August 2021 when the field contained a mature crop of maize (taken at 8 randomly selected locations between the crops). This field has been annually ploughed and cropped with conventional management since 1995 (Berdeni et al., 2021). The typical fertiliser inputs to this field were detailed in Humphries (2020) (full details see SI Table S1). Based on “World Reference Base (WRB)”, this soil is a Cambisol, with a silt loam texture containing stone fragments derived from the underlying dolomitic limestone (Guest et al., 2022; Humphries et al., 2023; IUSS Working Group WRB, 2015). The soil pH was 7.5 and its organic matter content was 6.6%. The cation exchange capacity of this Spen Farm soil is ~ 19 meq/100 g (Deans et al., 2023). The soil, as collected, contained about 80 mg Cu and 110 mg Zn per kg dry soil solids. The soil and digested sludge were stored at 4 °C prior to any experiments.

#### Preparation of sludge-amended soil

Two sludge-amended soils were used in this combined study. In the rhizo-pot experiment, to monitor the effect of the root zone on Cu and Zn uptake, a sludge-amended soil with about 5000 mg/kg Cu and Zn was prepared (full details see Table S2). This concentration represents an agricultural soil heavily contaminated by sludge-introduced metals (it was chosen so that metal concentration could be readily determined by aqua regia digestion and ensure that most of the metals in the sludge-amended soil were introduced with the sludge). A previous investigation had determined that linear sorption behaviour of digested sludge was largely maintained up to 2 wt% of Cu and Zn (Feng et al., 2024). Therefore, in this experiment the collected original digested sludge was amended to a final concentration of ~ 20,000 mg/kg Cu and Zn by direct addition of analytical grade ZnCl_2_ and CuCl_2_·2H_2_O to the sludge (which is 95% water). Then the metal-amended sludge was added to the soil to produce a final ratio of 25% by dry weight. The amended sludge and soil were mixed for ~ 10 h in a Hobart mixer (model: A200) and then allowed to stand for 21 days. It is noted that the Zn and Cu concentrations in the metal-amended sludge were about 3–5 times higher than the US-EPA limits for Zn and Cu in sewage sludge (7500 mg/kg and 4300 mg/kg, respectively; (EPA, 2022)), but comparable with maximum metal concentrations in sludges that have applied to agricultural land in the past (Babel & del Mundo Dacera, 2006; Jain & Tyagi, 1992; Olthof & Lancy, 1979). However, these concentrations were not intended to replicate current or past agricultural practice, but to ensure the Cu and Zn added with the amended sludge dominated the Cu and Zn concentrations in the resulting sludge-amended soils, so that the changes in their spatial distribution in close proximity to plant roots could be investigated.

The sludge-amended soil used in the plug-tray experiment contained ~ 1000 mg/kg of both Cu and Zn. This concentration represents an agricultural soil moderately contaminated by sludge-introduced metals (it was chosen so that most of the metals in the sludge-amended soil were introduced with the sludge, and so that metal binding environment could be determined by XANES analysis). This sludge-amended soil was prepared for a preceding study, where it had undergone three rounds of spring barley growth. It was a mixture of the amended sludge and agricultural soil described above, but it contained 5% amended sludge by dry weight. The pH value of this sludge-soil mixture was 7.6 and organic matter content of this mixture was 7.5%. More details of the characterization of the original digested sludge, the amended sludge and the agricultural soil used in that study were shown in Feng et al. (2024).

### Plant growth chamber

All plant growth experiments were undertaken in the LMS Cooled Incubator. Three LED growth lamps (Knightsbridge UCLED9CW, 230 V, 9 W, LED 4000 K-538 nm) were mounted above the plants, on a 12-h on/off cycle. The lamps delivered about 99–107 and 43–48 μmol/m^2^/s of photosynthetically active radiation for the rhizo-pot experiment and the plug-tray experiment, respectively (measured at the level of rhizo-pot and plug-tray surfaces). A day/night temperature regime of 26 °C/24 °C was chosen to maximize the plant growth (GHEDirect, 2024). The growth conditions matched the experiment reported by Feng et al. (2024), as shown in SI Sect. S1.

### Rhizo-pot growth experiment

#### Establishment of rhizo-pot system

A rhizo-pot system similar to that described by Kissoon et al. (2010) was used in this experiment (see Fig. S2). It consisted of an upper plant compartment containing perlite and a lower soil compartment separated by a 32-μm nylon mesh. During plant growth phase, a horizontal root mat forms above the mesh. The nylon mesh physically separates the plant roots from the lower soil compartment but allow other substances such as water, nutrients and root exudates to exchange between the two compartments (Chen et al., 2021; Gao et al., 2014; McNickle & Godoy, 2020; Oburger et al., 2014). The lower compartment was loaded with 25 g sludge-amended soil containing ~ 5000 mg/kg Zn and Cu (moisture content of 32.0%, slightly compacted to a height of 20 mm). The moisture content of this sludge-amended soil was then adjusted to 35% (about 70–80% of the field capacity) and maintained at this level during growth phase by regular irrigation with distilled water. Three seedlings of spring barley were placed in the upper compartment (germination procedures see SI Sect. S2). 4.5 g of saturated perlite (soaked in distilled water for 1 h) was used to cover the plant seedlings. The upper compartment was then placed over the sludge-amended soil surface on the lower compartment (planted rhizo-pot). Control rhizo-pots were prepared in the same way as the “planted rhizo-pot”, but with no plant seedlings. Both planted and control rhizo-pots were placed in the incubator.

A total of 45 planted and 15 control rhizo-pots were prepared for this experiment, which was conducted over five rounds of plant growth, with a 6-week growth period in each round. Upon the completion of each round, all plants were harvested. After the first, third and fifth rounds, 5 planted and 3 control rhizo-pots were sacrificially sampled. In addition, after each round, the nylon meshes were replaced in all the remaining rhizo-pots and three more spring barley seedlings were grown in fresh perlite.

#### Sampling

**Plants**: plant height (from perlite surface to the tip of plant flag leaf) was recorded every 7 days during growth period. After each 6 weeks growth round, the plant roots system was carefully removed from the upper compartment, washed and oven-dried (washing and drying procedures see SI Sect. S3). Dry weight biomass of the plant roots and shoots were measured separately. The plant shoots and roots were then ground in a Retsch CryoMill and stored at 4 °C for further analysis.

**Soils**: for the first, third and fifth rounds, the sludge-amended soil in the sampled lower compartments was excavated in six sublayers (0–2, 2–4, 4–7, 7–10, 10–15 and 15–20 mm distance from the nylon mesh). The soil from each sublayer of each pot was thoroughly mixed separately, air-dried, disaggregated, and sieved with a 106-μm sieve for aqua regia digestion analysis.

#### Aqua regia digestion

Total metal concentrations in soils, sludges and plants were determined by aqua regia digestion. Specific steps of this digestion procedure were shown in SI Sect. S4. The precision of this aqua regia digestion procedure for Cu and Zn has been determined to be < 7% (relative standard deviation of three measurements on samples), with an accuracy of 95–105% (percent recovery from certified materials), meeting the criteria for satisfactory precision (≤ 20%) and accuracy (80–120%) in aqua regia digestion (Chen & Ma, 2001).

#### Statistical analysis

Before conducting statistical analysis, all the data were assessed for normality and homogeneity of variance. Statistical analysis of Cu and Zn concentrations in the sublayers of planted and control soil was performed by one-way analysis of variance (ANOVA) at a 5% significance level using SPSS version 26.0. Post hoc tests (Least Significant Difference, LSD) were undertaken after ANOVA. The Student’s *t*-test was used to determine the significant level wherever required.

### Plug-tray growth experiment

#### Establishment of plug-tray system

Plug-trays containing square prismatic “pots” (top surface: 2 cm × 2 cm; bottom surface: 1 cm × 1 cm; height: 3 cm) were used for this experiment. The drainage hole at the pot bottom was covered with two pieces of needle-punched polypropylene geotextile (supplied by Spudulica) to avoid soil loss. A close-fitting lining of aluminium mesh (supplied by ISOPON) was then inserted as the permeable sample holder. Each pot was filled with 7.5 g sludge-amended soil (~ 1000 mg/kg Cu and Zn) with an initial moisture content of 11.6%. One spring barley seedling was planted in each planted pot. The moisture content of the sludge-amended soil was adjusted to 35% and maintained at this moisture content during growth phase. The plants were allowed to grow for a period of 6 weeks, then the plant shoots were harvested, and the planted pots were prepared for later analysis.

#### Resin impregnation of the soil and roots

The sludge-amended soil containing the plant roots was transferred to transparent polypropylene plastic cylindrical containers by carefully lifting the aluminium mesh holder from the plug-tray pots. A fluid displacive drying and the resin-impregnating procedure, developed from EMS (2024), was used to preserve the rootlet zone within the sludge-amended soil pots with minimal physical disturbance. Details of this protocol are given in SI Sect. S5.

Before the plug-tray growth experiment, a sample of the sludge-amended soil was also prepared for determining the initial binding environments of Cu and Zn (for comparison with the metal binding environment in the rhizosphere and within the root structures). This sludge-amended soil was dried by solvent displacement and then embedded in the 30-mm epoxy resin blocks (EpoThin 2, a glycidyl ether epoxy resin). The blocks were ground and polished as described above.

#### Scanning electron microscopy data acquisition

Polished samples were carbon-coated and observed with a scanning electron microscope-energy dispersive X-ray spectroscopy (SEM–EDS) as an initial assessment of spring barley root structures and metal distribution patterns. SEM–EDS operating conditions were shown in SI Sect. S6.

#### EPMA data acquisition

Elemental mapping of the carbon-coated samples was performed on a JEOL JXA-8230 Electron Probe Microanalyzer operating at 20 keV incident energy. This instrument captures secondary and backscattered electron images. It is equipped with an Energy Dispersive Spectrometer (EDS) and 5 Wavelength Dispersive Spectrometers (WDS), allowing the simultaneous analysis of different elements and the generation of distribution maps for each element. The spatial resolution of the elemental maps is about 1 μm.

#### μXRF and μXANES data acquisition

μXRF element maps and μXANES spectra were collected on beamline I18 at the Diamond Light Source. Operating conditions were detailed in SI Sect. S7. Multielement μXRF spectra were collected from regions that were approximately 0.5 × 0.5 mm. These where then processed with the beamline software in real time to produce elemental maps. Based on μXRF elemental maps, spots of interests were selected for μXANES analysis (see SI Fig. S6). Only single μXANES spectra were collected from any one spot within a sample (to minimize beam damage, limit scans to a maximum of 5–10 min on any one spot), and then the sample stage automatically moved to expose an unaffected part of the sample before subsequent scans. Collected μXANES spectra were normalised in Athena version 0.9.26 over full data range (Ravel & Newville, 2005). Normalised spectra were then corrected for any drift in E_0_ using the data collected from Cu- and Zn- metal reference spectra. Linear combination fitting (LCF) analysis was performed using the selected reference standards (full details of reference standards see Fig. S5, Tables S5 and S6) to determine the most likely combinations that best fits the sample spectra. The number of reference standards permitted in each fit was limited to 3 to reduce the degree of freedom. It is noted that LCF analysis often cannot identify the exact phases present in environment samples as many phases can share similar molecular coordination environments, but it provides valuable information on the valence states and dominant elemental binding environments present.

## Results

### Rhizo-pot experiment results

#### Plant analysis

**Fig. 1:**
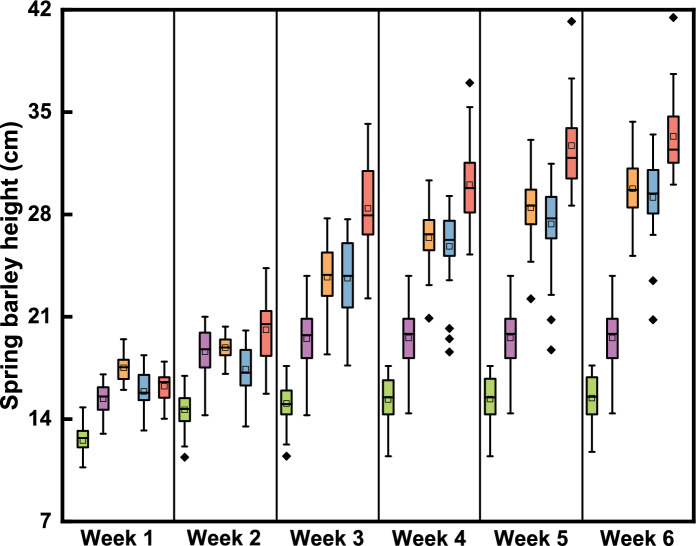
The recorded height of spring barley during each growth period in the rhizo-pot experiment (green: 1st round; purple: 2nd round; yellow: 3rd round; blue: 4th round; pink: 5th round. shaded boxes show the median values and interquartile range; tails indicate 1.5 × IQR; □: mean value; ♦: outliers)

During the first growth round, the plant growth rate was slower than in the subsequent growth rounds, and there was little change in plant height after 3 weeks (see Fig. 1). The growth rate was slightly higher in the second round, but again there was little change in plant height after 3 weeks. Growth in the third, fourth and fifth rounds was similar, with a higher initial growth rate than in the earlier rounds, and with growth continuing over the 6-week growth period. As a result, the average plant height after 6 weeks was 15.4 cm, 19.6 cm, 29.7 cm, 29.2 cm, and 33.3 cm in five growth rounds. At the end of each growth period, the dry weight biomass of per plant were 0.04 g/plant, 0.04 g/plant, 0.09 g/plant, 0.10 g/plant and 0.14 g/plant, respectively (see Table 1). The dry weight biomass ratio of shoots to roots was approximately 2 in all growth rounds.

**Table 1:** Dry weight biomass of spring barley roots and shoots

Growth round	Dry weight biomass of shoots (g)	Dry weight biomass of roots (g)	Survived planted pots number	Harvested plants number	Dry weight biomass of each plant shoot (g/plant)	Dry weight biomass of each plant root (g/plant)
1st round	4.37	1.23	42	126	0.03	0.01
2nd round	2.94	1.27	32	96	0.03	0.01
3rd round	5.91	3.23	32	96	0.06	0.03
4th round	5.22	3.05	27	81	0.06	0.04
5th round	7.22	4.24	27	81	0.09	0.05

**Fig. 2:**
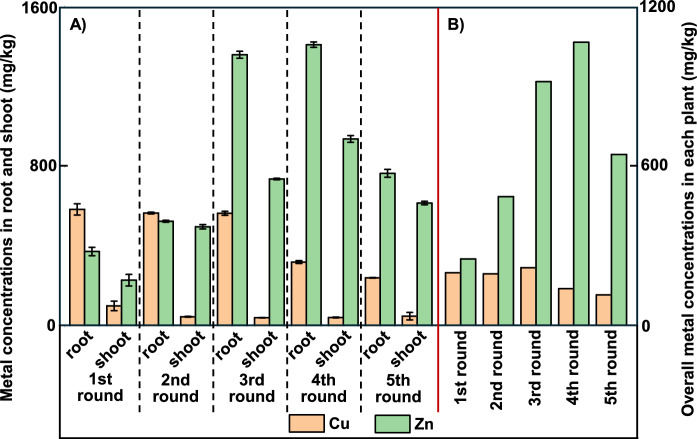
**A** Zn and Cu concentrations in spring barley roots and shoots of five growth rounds and **B** overall Zn and Cu concentrations in each plant for five growth rounds in the rhizo-pot experiment

The average Cu concentration in the spring barley was about 210 mg/kg in the first three growth rounds, decreasing to 150 and 120 mg/kg in the fourth and fifth growth rounds (see Fig. 2). Interestingly, the Cu was partitioned strongly to plant roots, where the average Cu concentration was about 570 mg/kg in the first three growth rounds, decreasing to about 320 and 240 mg/kg in the fourth and fifth growth rounds (the average ratio of roots to shoots for Cu concentrations was ~ 8). The average Zn concentration in the spring barley increased in the first four growth rounds, before decreasing in the fifth growth round (260 mg/kg, 510 mg/kg, 960 mg/kg, 1110 mg/kg and 670 mg/kg, respectively). The Zn concentration in the roots followed same pattern over the five growth rounds (370 mg/kg, 530 mg/kg, 1360 mg/kg, 1400 mg/kg and 770 mg/kg respectively), but Zn partitioned less strongly to the plant roots than Cu (the average ratio of roots to shoots for Zn concentrations was ~ 1.5).

#### Cu and Zn concentrations in the rhizo-pot soil sublayers

One-way ANOVA indicated that there were no significant differences in Cu concentrations between 0 and 20 mm control soil sublayers after the first round (*p* < 0.05; see Table 2). Whereas for both the third and fifth rounds, Cu concentrations in the 0–2 mm control soil sublayers were significantly lower than the 4–20 mm sublayers. After these rounds the Cu concentration in the 2–4 mm sublayer was intermediate between the 0–2 mm and 4–20 mm layers, but not statistically different from either. In the planted soils, after the first and third rounds, one-way ANOVA indicated that Cu concentrations in the 0–2 mm sublayer were significantly lower than 4–20 mm sublayers. As seen in the control, the Cu concentration in the 2–4 mm sublayer of the planted soil was intermediate between the 0–2 mm and 4–20 mm sublayers, but not statistically different from either. After five growth rounds, Cu concentrations in the 0–4 mm sublayers of the planted soil were significantly lower than the 4–20 mm sublayers. Also, the Cu concentration in the 0–2 mm sublayer was significantly lower than in the 2–4 mm sublayer. The Cu concentrations in 0–2 mm and 2–4 mm sublayers of the planted soils were in each case lower than in the equivalent control sublayers, but only one of the six comparisons passed the Student’s *t* test for significance (*p* < 0.05). This suggests that the observed reduction in Cu concentration in the near surface soils was not directly associated with plant growth, and that 5 rounds of plant growth had little influence on Cu concentration in adjacent soil at the mm scale of this experiment.

**Table 2:** Mean Cu concentrations at different sublayers of control and planted rhizo-pot soils after first, third and fifth growth rounds (mg/kg of dry soils)

Sublayers (mm)	1st round			3rd round			5th round		
	Control	Planted	*t* test *p* value	Control	Planted	*t* test *p* value	Control	Planted	*t* test *p* alue
0–2	4839 ± 150^ab^	4705 ± 58^c^	0.259	4892 ± 76^c^	4708 ± 80^c^	**0.019**	4731 ± 75^c^	4654 ± 89^c^	0.259
2–4	4797 ± 92^ab^	4751 ± 52^bc^	0.390	4984 ± 109^bc^	4861 ± 73^bc^	0.100	4835 ± 130^bc^	4815 ± 112^b^	0.824
4–7	4900 ± 67^ab^	4913 ± 104^a^	0.857	5039 ± 68^ab^	5000 ± 188^ab^	0.745	4988 ± 83^ab^	5012 ± 57^a^	0.642
7–10	4944 ± 103^a^	4849 ± 58^ab^	0.138	5097 ± 51^ab^	5080 ± 241^a^	0.907	5106 ± 49^a^	5122 ± 82^a^	0.769
10–15	4900 ± 12^ab^	4930 ± 104^a^	0.560	5150 ± 66^a^	5051 ± 85^ab^	0.138	5095 ± 46^a^	5100 ± 184^a^	0.967
15–20	4725 ± 86^b^	4902 ± 87^a^	**0.031**	5085 ± 83^ab^	5024 ± 77^ab^	0.332	5146 ± 195^a^	5082 ± 49^a^	0.489

Values are expressed as mean ± standard deviation (*n* = 3 for control soils and *n* = 5 for planted soils). One-way ANOVA has been applied to each column of data (*p* < 0.05). Different superscript letters indicate a significant difference in mean Cu concentration between each sublayer for that test condition. For example, a population labelled ^a^ is significantly different from ^b^ or ^c^, while ^ab^ would not be significantly different from a population annotated as ^a^ or ^b^, but would be significantly different from those labelled ^c^. The Students’ *t* test was used to determine the significance of the difference in mean Cu concentration between the sublayers of planted soil and control soil in each growth round (see the bold *p* value)

The Bold numbers suggest there are significant difference in mean Cu concentration between the sublayers of planted soil and control soil in each growth round

For Zn, one-way ANOVA showed that there were no significant differences between control soil sublayers after any of the growth rounds (*p* < 0.05; see Table 3). In the planted soils, Zn concentrations after the first round were significantly lower in the 0–2 mm sublayer than in the 2–20 mm sublayers, with little difference between the other sublayers. After three rounds, the Zn concentration in the 0–2 mm sublayer was significantly lower than in the 2–4 mm sublayer, which in turn was significantly lower than the Zn concentrations the 4–20 mm sublayers. After five rounds, the Zn concentrations in the 0–2 mm and 2–4 mm sublayers were comparable, but both were significantly lower than the Zn concentration in the 4–7 mm sublayer, which in turn was significantly lower than the Zn concentration in the underlying sublayers. The Zn concentrations in the 0–2 mm and 2–4 mm sublayers of the planted soils were in each case lower than in the equivalent control sublayers, and for five comparisons this difference was significant (the Student’s *t* test; *p* < 0.05). This suggests that the observed reduction in Zn concentration in the near surface soils was enhanced by the plant growth.

**Table 3:** Mean Zn concentrations at different sublayers of control and planted rhizo-pot soils after first, third and fifth growth rounds (mg/kg of dry soils)

Sublayers (mm)	1st round			3rd round			5th round		
	Control	Planted	*t* test *p* value	Control	Planted	*t* test *p* value	Control	Planted	*t* test *p* value
0–2	4824 ± 19^bc^	4579 ± 110^c^	**0.010**	4754 ± 257^b^	4085 ± 82^d^	**0.001**	4667 ± 151^a^	3635 ± 347^c^	**0.003**
2–4	4976 ± 54^abc^	4834 ± 32^b^	**0.003**	4943 ± 46^ab^	4563 ± 119^c^	**0.001**	4693 ± 378^a^	3970 ± 473^c^	0.067
4–7	5041 ± 71^abc^	4987 ± 49^ab^	0.242	4903 ± 163^b^	4926 ± 394^b^	0.928	4695 ± 389^a^	4605 ± 165^b^	0.655
7–10	5088 ± 156^ab^	5043 ± 167^a^	0.718	4867 ± 209^b^	5184 ± 264^ab^	0.129	4842 ± 499^a^	5015 ± 161^a^	0.483
10–15	5104 ± 90^a^	5066 ± 82^a^	0.559	5045 ± 186^ab^	5245 ± 138^a^	0.129	4821 ± 127^a^	5056 ± 273^a^	0.219
15–20	4798 ± 286^c^	4929 ± 203^ab^	0.471	5259 ± 70^a^	5046 ± 74^ab^	**0.007**	5049 ± 215^a^	5079 ± 64^a^	0.773

Values are expressed as mean ± standard deviation (*n* = 3 for control soils and *n* = 5 for planted soils). One-way ANOVA has been applied to each column of data (*p* < 0.05). Different superscript letters indicate a significant difference in mean Zn concentration between each sublayer for that test condition. For example, a population labelled ^a^ is significantly different from ^b^ or ^c^, while ^ab^ would not be significantly different from a population annotated as ^a^ or ^b^, but would be significantly different from those labelled ^c^. The Students’ *t* test was used to determine the significance of the difference in mean Zn concentration between the sublayers of planted soil and control soil in each growth round (see the bold *p* values)

The Bold numbers suggest there are significant difference in mean Zn concentration between the sublayers of planted soil and control soil in each growth round

### Plug-tray experiment results

#### Cu and Zn elemental distribution

**Fig. 3:**
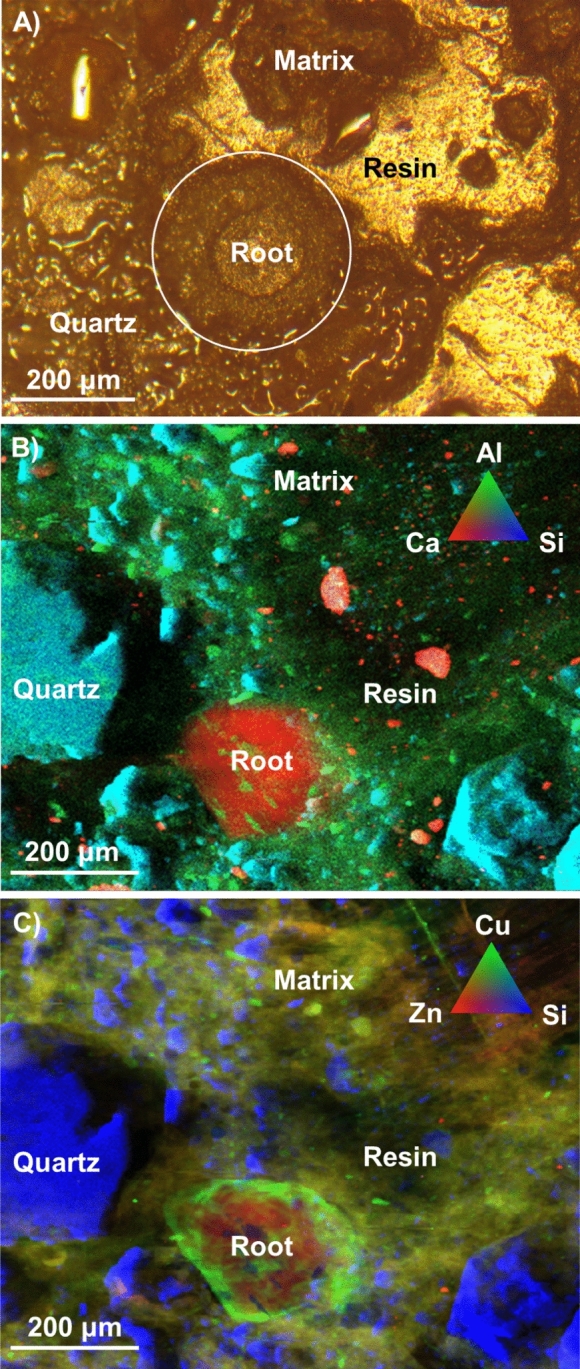
**A** Optical image cross section of resin embedded spring barley root grown in sludge-amended soil showing location of a plant root. μXRF false colour maps showing distribution of **B** Si–Al–Ca, and **C** Si–Cu–Zn in close proximity to the root of (A). Note: there is a small difference in the frame of reference for optical microscopy and μXRF mapping

**Fig. 4:**
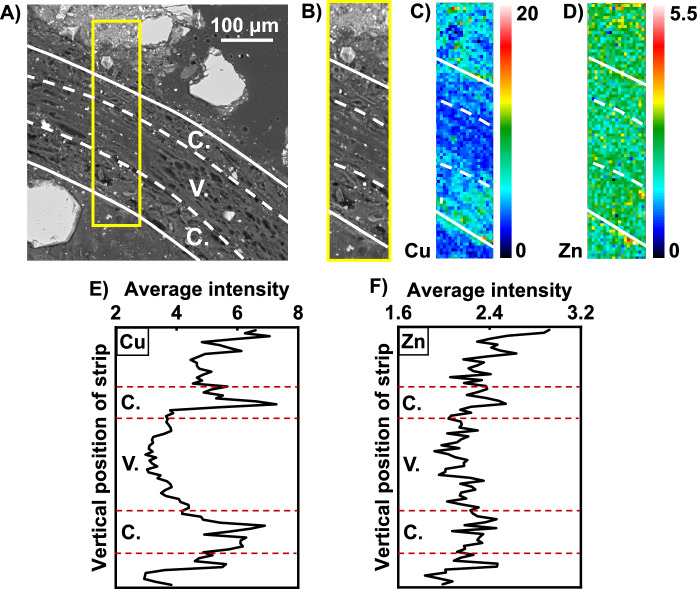
Distribution of Cu and Zn in the vicinity of a spring barley root (longitudinal section). **A** EPMA backscattered electron image; **B** the subregion of the image selected for EPMA analysis; **C** Cu and **D** Zn maps of the selected region; **E** and **F** average intensity within the selected region as a function of vertical position. Labelling: C.—cortex; V.—vascular structure

The resin-impregnated soil samples from the plug-tray experiment were viewed under an optical microscope, and locations where spring barley roots were orientated roughly perpendicular and roughly parallel to the polished plane were identified for subsequent analysis. When a root is perpendicular to the polished plane (see Fig. 3), it is visible on the μXRF Si-Al-Ca map as a “Ca” hot spot. The Si–Cu–Zn µXRF map of the same location showed that Cu was present throughout the root structures, but with higher relative concentrations in the outer cortex. Small Cu-rich spots were seen distributed in the vicinity of the root surfaces. A similar Cu distribution pattern was observed in the longitudinal section of a different root imaged by EPMA (see Fig. 4). The element map suggests that the Cu concentration is higher near the root surface and within the root outer cortex, than within the vascular region of the root (see Fig. 4C). This pattern is clearer when pixels within the same horizontal row of the strip map are averaged (see Fig. 4E), and suggests that the Cu concentration within the root outer cortex is higher than it is outside the root (immediately adjacent to the root surfaces).

The Si–Cu–Zn µXRF elemental maps showed that Zn concentrations were similar in the root outer cortex and inner vascular region. Like Cu, small Zn-rich spots were found in the rhizosphere (also seen in the SEM elemental maps shown in Fig. S4). The average intensity within the vertical strip map across a root orientated parallel to polished plane being imaged showed that Zn concentrations were similar in the root (cortex and vascular tissues) and rhizosphere soil immediately adjacent to the root.

#### Cu and Zn speciation

**Fig. 5:**
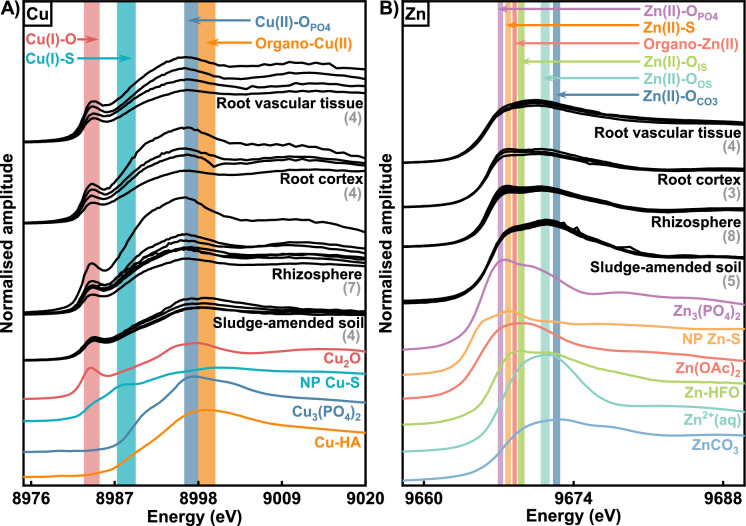
K-edge μXANES spectra for **A** Cu and **B** Zn collected from different spots of interests based on μXRF elemental map of Fig. 3 and XANES spectra of selected reference standards. The number in grey brackets is the number of spot analyses shown. The coloured bands are provided to guide the eye to significant spectra feature present in reference standards spectra

The Cu μXANES spectra collected from different locations within Fig. 3 showed that the Cu bonding environments varied between the plant root structures, the rhizosphere and the sludge-amended soil (see Fig. 5; locations from which the spectra were collected are shown in SI Fig. S6). LCF results indicate that the three most abundant Cu bonding environments in the sludge-amened soil (prior to plant growth) were Cu(I)-O (~ 45%), Cu(I)-S (~ 30%) and organo-Cu(II) (~ 25%), as shown in Table 4. However, neither Cu(I)-S nor organo-Cu(II) were among the three most abundant bonding environments in either the rhizosphere or the root structures. The dominant Cu bonding environments in the rhizosphere were Cu(I)-O (~ 60%) and Cu(II)-O_PO4_ (~ 40%, absorbed/bonded to phosphate), with similar proportions of Cu in these two bonding environments in the root cortex. The same two bonding environments were also found in the vascular tissue, but with a larger proportion of Cu(I)-O (~ 70%) and a lower proportion of Cu(II)-O_PO4_ (~ 30%).

**Table 4:** Relative abundance of Cu species determined from LCF analysis of Cu μXANES spectra of samples

Samples	Spots	Cu species (%)				*R*-factor
		Cu_2_O	Cu-HA	NP Cu–S	Cu_3_(PO_4_)_2_	
Sludge-amended soil	1	44 (5)	25 (2)	31 (6)	–	0.0137
	2	42 (4)	26 (5)	33 (4)	–	0.0086
	3	49 (6)	26 (13)	25 (6)	–	0.0162
	4	44 (2)	23 (5)	33 (2)	–	0.0028
	Average	45	25	30	**–**	**–**
Rhizosphere	5	52 (10)	–	–	48 (12)	0.2313
	6	49 (9)	–	–	51 (14)	0.1783
	7	7 (25)	–	–	93 (25)	0.6840
	8	64 (5)	–	–	36 (5)	0.0802
	9	75 (9)	–	–	25 (5)	0.0742
	10	79 (2)	–	–	21 (5)	0.0148
	11	79 (8)	–	–	21 (6)	0.1195
	Average	58	**–**	**–**	42	**–**
Root cortex	12	84 (3)	–	–	16 (3)	0.0237
	13	64 (9)	–	–	36 (9)	0.1830
	14	31 (17)	–	–	69 (16)	0.4020
	15	66 (6)	–	–	34 (6)	0.1079
	Average	61	**–**	**–**	39	**–**
Root vascular tissue	16	65 (9)	–	–	35 (9)	0.1797
	17	49 (14)	–	–	51 (14)	0.3508
	18	80 (5)	–	–	21 (5)	0.0650
	19	91 (2)	–	–	9 (5)	0.0139
	Average	71	**–**	**–**	29	**–**

LCF derived errors are given in parentheses

The selected spots of interests for Cu analysis are shown in Fig. S6. Cu–HA, Cu-humic complex; NP Cu–S, Cu(I)-S nano particles

LCF of Zn μXANES spectra indicated that the dominant Zn bonding environments in the sludge-amended soil, rhizosphere and plant structures were also different (see Fig. 5 and Table 5). In the sludge-amended soil, Zn was predominantly found in the bonding environments similar to that in inner sphere complexes with metal oxides (Zn(II)-O_IS_, ~ 70%), aqueous Zn^2+^ (which is indistinguishable from outer sphere bonding complexes; Zn(II)-O_OS_, ~ 20%) and Zn absorbed/bonded to phosphate (Zn(II)-O_PO4_, ~ 10%). The dominant Zn bonding environments in the rhizosphere were absorbed to/incorporated into carbonates (Zn(II)-O_CO3_, ~ 40%), Zn(II)-S (~ 35%) and Zn(II)-O_IS_ (~ 25%). In the root structures, the dominant bonding environments were Zn(II)-O_CO3_, Zn(II)-S and Zn acetate complexes (organo-Zn(II)). In the root cortex, the relative proportions were ~ 55%, ~ 30%, and ~ 15%, respectively, while in the vascular tissue they were ~ 50%, ~ 20% and ~ 30%.

**Table 5:** Relative abundance of Zn species determined from LCF analysis of Zn μXANES spectra of samples

Samples	Spots	Zn species (%)						*R*-factor
		Zn_3_(PO_4_)_2_	Zn^2+^ aq	Zn–HFO	NP Zn–S	ZnCO_3_	Zn(OAc)_2_	
Sludge-amended soil	1	16 (4)	16 (2)	68 (2)	–	–	–	0.0028
	2	9 (2)	13 (2)	77 (3)	–	–	–	0.0041
	3	9 (7)	24 (3)	67 (3)	–	–	–	0.0054
	4	13 (1)	29 (2)	58 (7)	–	–	–	0.0038
	5	11 (5)	15 (2)	74 (2)	–	–	–	0.0029
	Average	12	19	69	**–**	**–**	**–**	**–**
Rhizosphere	6	–	–	19 (8)	35 (12)	46 (7)	–	0.0968
	7	–	–	18 (8)	31 (16)	51 (12)	–	0.0997
	8	–	–	40 (14)	36 (5)	25 (5)	–	0.0567
	9	–	–	29 (7.0)	37 (13)	35 (6)	–	0.0714
	10	–	–	32 (7)	30 (11)	38 (6)	–	0.0691
	11	–	–	34 (7)	33 (7)	33 (6)	–	0.0649
	12	–	–	20 (8)	36 (16)	44 (17)	–	0.0938
	13	–	–	22 (8)	39 (6)	39 (12)	–	0.0876
	Average	**–**	**–**	27	35	39	**–**	**–**
Root cortex	14	–	–	–	43 (5)	57 (9)	0 (0)	0.1164
	15	–	–	–	34 (9)	52 (13)	14 (8)	0.0834
	16	–	–	–	21 (8)	52 (12)	27 (7)	0.5955
	Average	**–**	**–**	**–**	33	54	14	**–**
Root vascular tissue	17	–	–	–	24 (5)	53 (10)	23 (5)	0.0264
	18	–	–	–	18 (5)	50 (10)	32 (4)	0.0221
	19	–	–	–	12 (4)	49 (9)	39 (4)	0.0152
	20	–	–	–	31 (7)	55 (4)	14 (8)	0.0531
	Average	**–**	**–**	**–**	21	52	27	**–**

LCF derived errors are given in parentheses

The selected spots of interests for Zn analysis are shown in Fig. S6. Zn^2+^ aq, aqueous Zn^2+^; Zn–HFO, Zn(II)-hydrous ferric oxide; NP Zn–S, Zn(II)S nano particles

## Discussion

### Macro-scale mobilization of contaminant metals from sludge-amended soil

In the rhizo-pot experiment, Zn was removed from near surface soil layers in both the planted pots and the control pots. However, at all three sampling times, Zn removal from the sludge-amended soil to a depth of 4 mm was significantly greater in the presence of plants than in the controls, with the amount of Zn removed increasing with number of growth rounds. After 5 rounds, 28% of the Zn in the surface layer was removed in the planted pots whereas 7% was removed in the controls.

Cu was also removed from the near surface soil layers in both the planted pots and the control pots. However, whilst the average Cu concentration in the planted pots was lower than that in the associated controls at all three sampling points in both the 0–2 mm and 2–4 mm soils, this difference was not large enough to be statistically significant. After 5 rounds, 10% of the Cu in the surface layer was removed in the planted pots and 9% was removed in the controls. The Cu uptake per plant was only about 25% of the Zn uptake, which may partially explain why Cu removal by the plants was not statistically significant from the control. The distance from the roots over which metal removal occurs may also be a factor. Previous work on the sludge-amended soil used in the plug-tray experiment found that the Cu concentration in soil particles on the surface of plant roots was significantly lower than the average value in the root zone, whereas the Zn concentration in the soil at the root surface was similar to the average value in the root zone (Feng et al., 2024). This suggests that the Cu uptake comes from sludge-amended soil in relatively close proximity to the roots (<< 1 mm), whereas Zn can be taken up from a larger region of the root soil (> 1 mm). The differences in metal removal behaviour probably reflect the differences in Zn and Cu speciation in the sludge-amended soil.

The average Zn μXANES LCF results for the sludge-amended soil used in the plug-tray experiment showed that Zn was present as Zn(II) adsorbed to metal oxide surfaces in inner-sphere and outer-sphere Zn(II) complexes, as well as a small amount as Zn(II) absorbed/bonded to phosphate. This is broadly consistent with previous bulk XANES data on this sludge-amended soil (which was prepared for a study that preceded this work). The bulk XANES identified the Zn coordination environments as Zn(II) adsorbed to metal oxide surfaces in both inner-sphere (70–80%) and outer-sphere (20–30%) complexes (Feng et al., 2024). It is likely that the Zn bonding environments in the sludge-amended soil of the rhizo-pot experiment will have been similar to that of the plug-tray experiment, although the higher amended-sludge loading in the rhizo-pot experiment may have affected the exact proportions of the two dominant bonding environments. As long-range (mm scale) interaction between plant roots and contaminant metals will only occur by diffusion (outward diffusion in plant exudates, inward diffusion of contaminant metals), it is suggested that the Zn removal from the rhizosphere soil created a concentration gradient that resulted in the Zn diffusion from the bulk soil to the rhizosphere. Also, it is likely that the Zn removal is from the pool of more bioavailable outer-sphere adsorbed Zn(II) ions.

The average Cu μXANES LCF results for the sludge-amended soil used in the plug-tray experiment showed Cu in a coordination environment characteristic of Cu(I) oxides, Cu(I) sulphides, and Cu(II)-organic complexes. This is also consistent with the previous bulk XANES data, which identified Cu bonding environments in the same sample as Cu(I) oxides (40–50%), Cu(I) sulphides (20–30%), and Cu(II)-organic complexes (30–40%) phases (Feng et al., 2024). It is likely that the Cu bonding environments in the sludge-amended soil of the rhizo-pot experiment will have been similar to that seen in the plug-tray experiment. Generally, Cu in the above three coordination environments has limited mobility in the sludge-amended soil, which is probably why very little Cu removal was observed at mm scale. The modest removal in both the planted and control soils was probably the result of irrigation with distilled water (a small proportion of Cu(II)-organic complexes may have been associated with soluble soil organics such as fulvic acids; Wu et al. (2002)).

### Cu and Zn speciation in rhizosphere soils

Cu in the rhizosphere soil was predominately in bonding environments characteristic of Cu(I) oxides (seen in the sludge-amended soil) and Cu(II) bonded to/incorporated into phosphates (not detected in the sludge-amended soil). The increase in the proportion of Cu in Cu(I) oxides relative to the bulk sludge amended soil may reflect the removal of other Cu phases close to plant roots, whilst the absence of Cu(I) sulphides and Cu(II)-organic complexes suggests that Cu initially in these bonding environments is either taken-up by the plant roots, or converted to Cu(II) phosphates. The presence of Cu(II) bonded to phosphate may suggest that the complexation of Cu(II) by P-containing ligands is an important process in the rhizosphere. P is usually sparingly available in soils, so plants have evolved chemical and biological changes strategies for mobilising P in the rhizosphere (Ding et al., 2021; Shen et al., 2011). The formation Cu(II)-phosphate phases in the rhizosphere may result from a reaction with plant mobilised P.

Zn in the rhizosphere soil was in bonding environments characteristic of Zn(II) inner-sphere complexes with metal oxide surfaces, Zn(II) sulphides and Zn(II) bonded to/incorporated into carbonates. As neither Zn(II) sulphides and Zn(II) carbonates were observed in this sludge-amended soil, they must be due to the close proximity of the roots of growing plants. The presence of Zn(II) bonded to/incorporated into carbonates is likely due to the reaction of Zn(II) with bicarbonate produced by plant and/or microbial metabolism. However, the reason for the Zn(II) sulphide-like bonding environment is less clear, but may be associated with changes in Cu chemistry in rhizosphere (conversion of Cu(I) sulphides to Cu(II)-phosphates). Nonetheless, formation of Zn(II) sulphide in the rhizosphere can promote Zn absorption by the plants for subsequent use in various metabolic reactions (Kanwal et al., 2022).

### Cu and Zn speciation in plant roots

Zn and Cu are both essential micronutrients for healthy plant growth, but both can disrupt plant metabolic processes at high concentrations (Behtash et al., 2022). Typically, plants require less Cu than Zn for healthy growth, and Cu becomes toxic to plants at lower concentrations (Behtash et al., 2022; Premier Tech Growers & Consumers, 2014). The rhizo-pot experiments showed that both Zn and Cu were taken up into the plants, with on-average ~ 4 × more Zn up-take per plant than Cu. Although Zn was far more readily translocated to the plant shoots (the ratio of roots to shoots concentrations was ~ 8 for Cu and ~ 1.5 for Zn), the average Zn concentrations in the roots were still about twice the average Cu concentrations. Feng et al. (2024) observed a similar pattern of lower Cu than Zn translocation when spring barley was grown directly in sludge-amended soil that contains only 20% of the Cu and Zn concentrations used in the rhizo-pot experiment. Others have reported similar results (Alam et al., 2002; Ali et al., 2004; Hilber et al., 2007). It is also interesting that the average ratio of Zn to Cu concentration in the plant shoots was ~ 9 over the 5 growth rounds. The recommended dietary allowance for adults is ten-fold higher for Zn (value) than Cu (value), and the tolerable upper intake level is 3–fivefold higher for Zn than Cu (EFSA, 2006; Government of Canada, 2024; NIH, 2022a, 2022b). Thus, similar additions of sludge-associated Cu and Zn to a soil may pose a similar risk to food crops where stem, leaves, flowers, and fruit are harvested. Further, whilst Zn concentrations were similar across the roots, Cu concentrations were higher in the root cortex than in the vascular tissue. One mechanism by which plants can respond to metal stress is to sequester them in the inactive tissues such as root epidermal cells (Cao et al., 2019; Wang et al., 2024). The resolutions of μXRF and EMPA cannot differentiate between the epidermis and the cortex, but it appears that spring barley has sequestered excess Cu, but not excess Zn, in the outer tissues of the root.

The Zn bonding environments in the root cortex differed from that seen in the rhizosphere. The root cortex did not contain Zn(II) in inner sphere complexes. Instead, Zn was found as Zn(II) absorbed to/incorporated into the carbonates, Zn(II) sulphides and Zn(II)-organic complexes. Plants acquire Zn from the sludge-amended soil solution, and transport it towards the root vasculature, primarily as aqueous Zn(II), but also as Zn-chelates (Zn(II)-organic complexes) (Balafrej et al., 2020; Broadley et al., 2007; Stanton et al., 2022). It is not possible to comment on the bonding environment of mobile Zn, as mobile species can be lost during resin impregnation. However, the presence of Zn(II) absorbed to/incorporated to carbonates is likely to have resulted from a reaction with bicarbonate in the roots. Thiol moieties (R-SH) in amino acids such as cysteine may account for Zn(II) sulphide in the cortex, as cysteine residues have a high affinity for aqueous Zn(II) ions (Zn(II)-cysteine complexes are critical mediators/precursors in protein synthesis present ~ 9% of the eukaryotic proteome) (Pace & Weerapana, 2014; Stanton et al., 2022; Zeng et al., 2011). The same Zn bonding environments were present in the vascular tissue as that seen in the cortex, but there is a slightly higher proportion of Zn(II)-organic complexes (at the expense of Zn(II) sulphides). This may be because Zn transport from roots to shoots in the xylem involves predominantly as Zn(II)-organic complexes (Al Jabri et al., 2022; Rizwan et al., 2019; Saifullah et al., 2016), probably as chelate complexes with nicotianamine, amino acids, or organic acids (Balafrej et al., 2020; Clemens et al., 2013). Nandal and Solanki (2021) also reported that the mobility of Zn in the plants occurs via complexation with organic compounds in xylem fluids.

The Cu bonding environments in the root cortex were similar to that seen in the rhizosphere (60% Cu(I) oxides and 40% Cu(II) absorbed/bonded to phosphate). In vascular tissue it was 70% Cu(I) oxides and 30% Cu(II) absorbed/bonded to phosphate. Others have also reported a high proportion of Cu as Cu(I) species in root tissues, and it has proposed that it is a mechanism by which plants detoxify Cu (Liu et al., 2019; Ryan et al., 2013; Sun et al., 2017). Cu is transferred within plants roots mainly as Cu(I)- or Cu(II)-complexes with chaperone proteins (Kumar et al., 2021; Wang et al., 2024). The μXANES LCF suggests that the majority of the Cu in the plant roots is immobile, which is supported by low Cu translocation to above ground structures, and localisation to the root cortex. Cell vacuoles are important plant organelles used to store mineral nutrients such as iron and phosphate (Peng & Gong, 2014; Wieczorek et al., 2022). Cu is also stored in vacuoles when absorbed in excess of nutritional needs (Peng & Gong, 2014). The μXANES data is compatible with Cu storage in cell vacuoles, particularly within the root cortex.

## Conclusion

The application of digested sewage sludge to agricultural soils offers a valuable source of nutrients and essential elements for plant production. However, it also introduces potential risks due to elevated concentrations of contaminant metals (particularly Zn and Cu). This work for the first time reveals that Zn introduced with digested sewage sludge is mobile on a mm scale, whereas Cu introduced with digested sewage sludge is only mobile on a sub-mm scale. Possibly, as a result, young spring barley plants took-up 4× more Zn than Cu even though these metals were present at similar concentrations in the sludge-amended soil. The speciation of Cu and Zn close to plant roots (i.e. in the rhizosphere soil) is different from that found more widely in sludge-amended soil, suggesting it is altered by plant growth. Local to the plant roots, Zn and Cu are in chemical environments that suggest their mobility has been reduced by the presence of plant roots. Zn that is taken-up by spring barley is relatively evenly distributed through the roots, with similar concentrations in the plant shoots, whereas most Cu that is taken-up is sequestered in the roots (particularly the root cortex), with only a small proportion translocated to above ground plant structures. These differences in the uptake of Cu and Zn by plants will be important in determining their relative risks (i.e. Cu is hazardous at lower concentrations than Zn, but Cu is trapped in the rhizosphere whereas Zn is translocated to plant shoots). Future work should focus on rhizosphere dynamics and crop-specific uptake to optimize sludge application, balancing nutrient recycling with environmental and crop safety concerns.

## Supplementary Information

Below is the link to the electronic supplementary material.

Supplementary file1 (DOC 12616 KB)

## Data Availability

No datasets were generated or analysed during the current study.
