# Supplementary material for: Distribution and speciation of Cu and Zn near spring barley (Hordeum vulgare) roots in digested sewage sludge-amended soil
Source: Environ Geochem Health. 2025 Apr 13;47(5):172. doi: 10.1007/s10653-025-02482-0 (PMC11994537; doi:10.1007/s10653-025-02482-0)
Supplement: Supplementary file 1 — Supplementary file1 (DOC 12616 KB) [file 10653_2025_2482_MOESM1_ESM.doc]

**Supplementary information**

**Distribution and speciation of Cu and Zn near spring barley (*Hordeum vulgare*) roots in digested sewage sludge-amended soil**

Jianting Feng ^a, b, *^, Ian T. Burke ^c^, Felipe E. Sepulveda Olea ^a^, Xiaohui Chen ^a^, Douglas I. Stewart ^a^

^a^ School of Civil Engineering, University of Leeds, Leeds LS2 9JT, UK

^b^ Key Laboratory of Ministry of Education for Geomechanics and Embankment Engineering, Hohai University, Nanjing 210024, Jiangsu, China

^c^ School of Earth and Environment, University of Leeds, Leeds LS2 9JT, UK

^*^ Corresponding author: [fjt18362982795@163.com](mailto:fjt18362982795@163.com)

**Consisting of**

7 sections

6 Figures

6 Tables

**Table S1 Fertiliser application record (2017-2019) obtained from** [**Humphries (2020)**](#_ENREF_6)

| Product | Date | Rate/ha | Units | Nutrient | Nutrient kg/ha |
| --- | --- | --- | --- | --- | --- |
| Boron Headland | 03/10/2017 | 0.49 | L | B | 0.07 |
| Manganese Headland | 03/10/2017 | 0.98 | L | Mn | 0.15 |
| Boron Headland | 13/10/2017 | 0.50 | L | B | 0.07 |
| Epso CombiTop | 13/10/2017 | 1.48 | kg | MgO | 0.19 |
|  |  |  |  | Mn | 0.06 |
|  |  |  |  | SO_3_ | 0.50 |
|  |  |  |  | Zn | 0.01 |
| Origin TSP | 08/11/2017 | 114.60 | kg | P_2_O_5_ | 52.72 |
| CF DoubleTop | 24/02/2018 | 168.42 | kg | N | 45.47 |
|  |  |  |  | SO_3_ | 0.50 |
| Origin MOP | 24/02/2018 | 62.20 | kg | K_2_O | 36.72 |
| Omex N26 + 5 (HH) | 07/04/2018 | 357.87 | L | N | 119.10 |
|  |  |  |  | SO_3_ | 22.90 |
| Omex N26 + 5 (HH) | 16/04/2018 | 191.37 | L | N | 63.69 |
|  |  |  |  | SO_3_ | 12.25 |
| Origin TSP | 01/08/2018 | 133.80 | kg | P_2_O_5_ | 63.67 |
| Omex N20+ 12.5 (HH) | 15/02/2019 | 286.30 | L | N | 71.92 |
|  |  |  |  | SO_3_ | 44.95 |
| Origin MOP | 22/02/2019 | 196.81 | kg | K_2_O | 117.72 |
| Manganese Headland | 01/04/2019 | 2.00 | L | Mn | 0.30 |
| Headland Cereal Plus | 01/04/2019 | 13.81 | L | MgO | 0.02 |
|  |  |  |  | Mn | 0.05 |
|  |  |  |  | Zn | 0.01 |
|  |  |  |  | Cu | 0.02 |
| Omex N26 + 5 (HH) | 08/04/2019 | 354.34 | L | N | 117.56 |
|  |  |  |  | SO_3_ | 22.61 |
| Headland Cereal Plus | 23/04/2019 | 11.51 | L | MgO | 0.02 |
|  |  |  |  | Mn | 0.04 |
|  |  |  |  | Zn | 0.01 |
|  |  |  |  | Cu | 0.02 |
| Epso CombiTop | 23/04/2019 | 1.58 | kg | MgO | 0.21 |
|  |  |  |  | Mn | 0.06 |
|  |  |  |  | SO_3_ | 0.54 |
|  |  |  |  | Zn | 0.02 |
| Omex N26 + 5 (HH) | 02/05/2019 | 158.03 | L | N | 52.43 |
|  |  |  |  | SO_3_ | 10.08 |
| Epso CombiTop | 24/05/2019 | 3.41 | kg | MgO | 0.44 |
|  |  |  |  | Mn | 0.14 |
|  |  |  |  | SO_3_ | 1.16 |
|  |  |  |  | Zn | 0.03 |
| Mag Super 80 | 24/05/2019 | 1.54 | L | MgO | 0.20 |

**Table S2** Cu and Zn concentrations in original digested sludge, original agricultural soil, metal-amended sludge, and sludge-amended soil used in the rhizo-pot experiment (mg/kg dry solids)

| Samples | Metal concentrations in samples (mg/kg) | |
| --- | --- | --- |
|  | Cu | Zn |
| Original digested sludge | 183 ± 2 | 600 ± 3 |
| Original agricultural soil | 78 ± 5 | 113 ± 5 |
| Metal-amended sludge | 20236 ± 18 | 18351 ± 151 |
| Sludge-amended soil | 5184 ± 76 | 5031 ± 60 |

Values are expressed as mean ± standard deviation (*n*=3)

# Section S1: Day/night temperature regime

In a previous study spring barley seedlings have been successfully planted in a sludge-amended soil for three growth rounds . In that study, the temperature inside the growth chamber was monitored by an auto-logger during growth phase (see Fig. S1). The average temperature of the growth chamber for day and night was ~26℃ and ~24℃, respectively. Based on the recorded temperature data, a day/night temperature regime of 26℃/24℃ was used in the combined study. This temperature regime was also recommended in the other source as it can provide a satisfactory growing environment for plants growth .

**Fig. S1** Temperature data inside a growth chamber for three growth rounds of spring barley . Part of temperature data of the first round was not monitored

**Fig. S2** Diagram of the rhizo-pot system. ID means inner diameter

# Section S2: Germination procedures of spring barley

The seedlings of spring barley were used in all plant growth experiments. Spring barley seeds (*Hordeum vulgare*, provided by Cotswold Seeds Ltd) were germinated between two filter papers soaked with half-strength Murashige and Skoog nutrient solution (2.2 g/L, pH=7.0) on petri dishes (5 seeds per dish). The petri dishes were sealed with parafilm and kept in the dark at room temperature for 2 days to allow seeds to germinate.

# Section S3: Washing and drying procedures of plants

All the plant roots were put into a 1-L beaker containing 600-700 mL distilled water, which was placed in an ultrasonic bath for 30 min to dislodge any perlite particles from the plant roots. The washing procedure was repeated 10 times. The plant shoots were also washed with distilled water. The washed plants were oven-dried at 105℃ for 30 min and then at 60℃ for a further 72 h. The dry weight biomass of plant roots and shoots were measured separately. Then the plant shoots and roots were ground in a Retsch CryoMill and stored at 4℃ for future analysis.

# Section S4: Aqua regia digestion procedures

***Determination of total metal concentrations in soil/sludge***: ~0.2 g of sample was weighed into a conical flask and a 10-ml aqua regia solution was added. The flask was heated on a hot plate (model: Cole-Parmer HP-200D-XL-C) to achieve effervescence and then for a further 30 min. After cooling, the contents of the conical flask were transferred to a 100-ml volumetric flask. Distilled water was added to a volume of 100 ml. This suspension was filtered (0.45-*μ*m syringe filter) and the solution was analysed by VARIAN 240 FS Atomic Absorption Spectrophotometer (AAS).

***Determination of total metal concentrations in plant roots/shoots:*** Plant matter reacts quickly with aqua regia, so to avoid excessive effervescence, the plant roots/shoots (~0.2 g) were first digested in hydrochloric acid (5 mL) at room temperature, and then evaporated to a dry residue. On cooling, this residue underwent aqua regia digestion, as described above.

To ensure comparability with other laboratories, total Zn and Cu concentrations in a certified reference material (LSKD-2, typical lake sediments from various locations within the Canadian Shield, provided by CCRMP, CANMET Mining and Mineral Sciences Laboratories) were determined by this aqua regia digestion procedure. The digestion results showed good agreement with the provisional reference values (see Table S3).

**Table S3** Comparison between total Zn and Cu concentrations in a certified reference material obtained by aqua regia digestion and provisional values

| Metals | Values of total metal concentration using aqua regia digestion (mg/kg) | Provisional values (mg/kg) | Recovery ratio  (%) |
| --- | --- | --- | --- |
| Cu | 36 ± 0 | 37 | 97 |
| Zn | 208 ± 5 | 209 | 100 |

Values are expressed as mean ± standard deviation (*n*=3)

# Section S5: Fluid displacive drying and resin-impregnating procedure

The sludge-amended soil containing the plant roots was transferred to transparent polypropylene plastic cylindrical containers by carefully lifting the aluminium mesh holder from the plug-tray pots. A fluid displacive drying and the resin-impregnating procedure, developed from [EMS (2024)](#_ENREF_3), was used to preserve the rootlet zone within the sludge-amended soil pots with minimal physical disturbance. This consisted of (1) Dehydration: immersion in a graded series of ethanol-water solutions (50%, 70%, 95% and 100% ethanol) was used for removal of aqueous pore fluids. Each step lasted one hour, and the 100% ethanol exchange was carried out twice; (2) Resin impregnation: a mixture of ethanol and fresh Spurr Resin in different proportions (2:1, 1:1, 1:3 and 100% fresh resin) was prepared and used successively for immersing samples (Spurr resin is a cycloaliphatic epoxy resin; formulations see Table S4; [Tegethoff and Briggman (2024)](#_ENREF_8)). Each step lasted for 24 hours, except for the step with 1:1 mixture, which lasted 48 hours. The 100% resin step was repeated three times to completely remove the residual ethanol; (3) Curing: the final resin-saturated samples were transferred to an oven for 12 hours at 70°C; (4) Cutting: the cured resin blocks were removed from plastic containers, and replicate samples were sectioned either vertically or horizontally, creating a set of blocks containing both transverse or longitudinal sections exposing the root-soil microfabric; (5) Grinding and polishing: the surface of each cut resin block was ground against silicon grinding papers of decreasing grit (P600 and P1200) before polishing with the 3-, 1- and ¼- 𝜇m water-free oil-based diamond paste (polished samples see Fig. S3).

**Table S4** Formulations of Spurr Resin

| Ingredients | Amount / g |
| --- | --- |
| ERL 4221 | 10 |
| Diglycidyl Ether of Polypropylene Glycol  (DER) | 8 |
| Nonenyl Succinic Anhydride  (NSA) | 25 |
| Dimethylaminoethanol  (DMAE) | 0.3 |

Note: the catalyst (DMAE) should be added last, after gently mixing the three other components

**Fig. S3** Longitudinal and transversal sections of samples after polishing treatment

# Section S6: SEM-EDS analysis

In the plug-tray experiment, the polished samples were carbon-coated and received a preliminary investigation of SEM-EDS on the metal distribution in the vicinity of the plant roots. The SEM is equipped with an Oxford Instruments X-max 150 SDD EDS using Aztec software. The SEM images of plant roots and the proximity to plant roots were obtained using the backscattered detector. The elemental mapping was performed at a resolution of 2 μm. The hot spots of interests in the elemental map were analysed by the equipped EDS to obtain the chemical composition.

After six weeks growth period in the sludge-amended soil, both cross section and longitudinal section of the plant roots were observed by SEM (see Fig. S4). It indicated that the size of spring barley root reached over hundreds micro-meters after 6 weeks growth. Both the inner vascular cylinder portion (mainly composed of pericycle, phloem, and xylem) and the outer cortex were observed. The root cortex was featured with the honeycomb-like structure.

**Fig. S4** SEM backscattered images of A) spring barley roots, B) cross section and C) longitudinal section of close proximity to plant roots. D) the sum spectrum of the sample A), B) and C) respectively. E) the spectrum of the three Zn-rich spots respectively (spots 1-3). Labelling: C. - cortex; V. - vascular tissue

# Section S7: μXRF and μXANES analysis

μXRF elemental and μXANES spectra data were collected at Cu and Zn K-edges (8979 and 9659 eV respectively) on beamline I18 at the Diamond Light Source operating at 3 GeV with a typical current of 200 mA, using a nitrogen cooled Si(111) double crystal monochromator and focussing optics. A pair of plane mirrors was used to reduce the harmonic content of the beam and the Kirkpatrick-Baez mirrors were used to produce either a focused or unfocused beam (0.005 mm or 0.1 mm diameter respectively at the sample) for microfocus or bulk sample analysis as required. For standards prepared as pressed pellets, K-edge spectra were collected in transmission mode using ionisation chamber detectors. For samples and solutions, data were collected in fluorescence mode using a 4 element 1 mm thick sensor Vortex Si Drifts detector with cube pre-amps. All data collection was performed at room temperature (~295 °K) within a He-filled bag (to lower the attentuation of low energy fluoressence X-rays). For the resin embedded blocks, approximately 0.5×0.5 mm multielment microfocus XRF spectra were collected. These where then processed by the beamline softeware in real time to produce elemental maps (Al, Ca, Cu, Zn and Si). Only single μXANES spectra was collected (~10 min) from any one spot within samples and the sample stage automatically moved to expose an unaffected part of the sample before subsequent scans. For bulk analysis, multiple scans were averaged to improve the signal to noise ratio using Athena version 0.9.26 . For all (μ)XANES spectra, absorption was normalised in Athena over the full data range and plotted. Spectra were corrected for any drift in E_0_ using the data collected from the metal foil standards. Linear combination fitting (LCF) was preformed in Athena using the full range of available standards to determine the most likely combinations of standards to best fit the sample. In the LCF analysis the number of standards used was limited to a maximum of 3 to reduce the degree of freedom present and all fits were forced by the software to produce results that summed to 100%. LCF typically produced results of elemental speciation with an uncertainity of ± 4%.

The sources or synthesis of all reference standards are listed in Table S5 and S6. All reference standards XAS spectra used in the LCF are shown in Fig. S5. All Cu reference spectra used were collected on Beamline I18 at the Diamond Light Source. Some of Zn-XANES spectra used were shared by the authors (Prof. Fred Mosselmans, Diamond Light Source; Prof Bryne Nygwena, University of Ediburgh; [Adele et al. (2018)](#_ENREF_1)); Zn(0)-foil data collected at the same time was also provided to ensure the correct calibration of standard spectra collected in different I18 sessions.

**Fig. S5** XANES spectra of reference standards of Cu and Zn

**Table S5** Reference standards label, chemical formula, and sources of reference compounds used for linear combination fitting analyses of Cu XANES data

| Reference standards sample (label/chemical formula) | Sources and/or synthesis method |
| --- | --- |
| Cu(II)CO_3_ | Purchased laboratory chemical |
| Cu(II)SO_4_ | Purchased laboratory chemical |
| Cu(II)O | Purchased laboratory chemical |
| Cu(I)_2_O | Purchased laboratory chemical |
| Covellite (Cu(I)S) | Natural mineral sample |
| Cu(II)-humic complex (Cu-HA) | 30 mL of 500 mg/kg Cu^2+^ (pH=3.28) and 3 g humic acid (Merck, UK) reacted for 24h, collected by centrifuging and dried at 40℃ |
| Cu^2+^_(aqueous)_ | 1000 mg/L Cu(NO_3_)_2_ solution |
| Cu(II)(CH_3_COO)_2_ | Purchased laboratory chemical |
| Cu(I)S nano particles (NP Cu-S) | Following the method of [Adele et al. (2018)](#_ENREF_1), 50 mL of 100 mM Cu^2+^ and 50 mL of 500 mM S^2-^ was reacted for 24h; separated solids recovered by centrifugation and dried at 20 ℃ in a 95%N_2_/5%H_2_ atmosphere |
| Cu(II)_3_(PO_4_)_2_ | Purchased laboratory chemical |
| Cu(II)(OH)_2_ | 10 ml of 6000 ppm Cu_2_(NO_3_)_2_ solution and 10 ml of 1M KOH reacted and collected by filtration |
| Cu(II)-hydrous ferric oxide  (Cu-HFO) | Hydrous ferric oxide synthesised according to the method of [Cornell and Schwertmann (2003)](#_ENREF_2) (containing haematite, goethite and ferrihydrite by XRD) was reacted with Cu^2+^ for 24 hours at pH 7-8. Solids recovered by filtration and dried at 20℃ |

**Table S6** Reference standards label, chemical formula, and sources of reference compounds used for linear combination fitting analyses of Zn XANES data

| Reference standards sample (label/chemical formula) | Sources and/or synthesis method |
| --- | --- |
| Zn(II)SO_4_ | Spectra shared by authors - [Adele et al. (2018)](#_ENREF_1) |
| Zn(II)O | Spectra shared by authors - [Adele et al. (2018)](#_ENREF_1) |
| Sphalerite (Zn(II)S) | Natural mineral sample |
| Zn(II)-FeOOH | Geothite synthesised according to the method of [Cornell and Schwertmann (2003)](#_ENREF_2) was reacted with Zn^2+^ for 24 hours at pH 7-8. Solids recovered by filtration and dried at 40℃ |
| Zn(II)S nano particles (NP Zn-S) | Spectra shared by authors - [Adele et al. (2018)](#_ENREF_1) |
| Zn(II)CO_3_ | Spectra shared by authors - [Adele et al. (2018)](#_ENREF_1) |
| Zn(II)(CH_3_COO)_2_ | Spectra shared by authors - [Adele et al. (2018)](#_ENREF_1) |
| Zn(II)_3_(PO_4_)_2_ | Spectra shared by authors - [Adele et al. (2018)](#_ENREF_1) |
| Zn^2+^ _(aqueous)_ | 1000 mg/L ZnCl_2_ solution |
| Zn(II)-humic complex (Zn-HA) | 30 mL 500 mg/kg Zn^2+^ (pH=3.28) and 3 g humic acid (Merck, UK) reacted for 24h, collected by centrifuging and dried at 40℃ |
| Zn(II)-hydrous ferric oxide (Zn-HFO) | Hydrous ferric oxide synthesised according to the method of [Cornell and Schwertmann (2003)](#_ENREF_2) (containing haematite, goethite and ferrihydrite by XRD) was reacted with Zn^2+^ for 24 hours at pH 7-8. Solids recovered by filtration and dried at 20℃ |
| Zn(II)(OH)_2_ | Precipitated from 6000 ppm ZnCl_2_ solution using 1 M KOH and collected by filtration and dried at 40℃ |

**Fig. S6** Spots of interests for Cu in A) the initial soil (before plug-tray growth experiment) and B) close proximity to the plant roots shown in Fig. 3. Spots of interest for Zn in C) the initial soil (before plug-tray growth experiment) and D) close proximity to the plant roots shown in Fig. 3

**References**
